# Supplementary material for: Non-linear response of summertime marine productivity to increased meltwater discharge around Greenland
Source: Nat Commun. 2018 Aug 14;9:3256. doi: 10.1038/s41467-018-05488-8 (PMC6092443; doi:10.1038/s41467-018-05488-8)
Supplement: Supplementary file 1 — Supplementary Information [file 41467_2018_5488_MOESM1_ESM.pdf]

Non-linear response of summertime marine productivity to increased meltwater discharge around Greenland

Hopwood et al.

Supplementary material

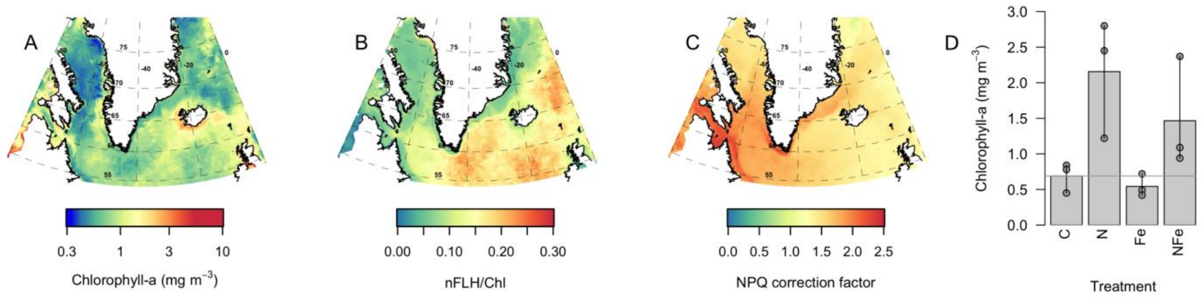

Supplementary Figure 1 Derivation of satellite-derived chlorophyll-a fluorescence quantum yields and experimental testing for limiting nutrients in Fram Strait. **(A)** Average chlorophyll-a concentrations (June, July, August; JJA), **(B)** Average JJA normalised fluorescence line height (nFLH) / chlorophyll-a concentration, **(C)** Non-photochemical quenching (NPQ) correction factor for fluorescence quantum yields, resulting from variable incident irradiance at the instant of nFLH capture [1] **(D)** A bottle-scale nutrient addition experiment from Fram Strait demonstrating NO_3 limitation of phytoplankton, as indicated by enhanced chlorophyll-a biomass following 48 h incubation with N (with, or without, added Fe) conducted as per [2]. Bars represent mean chlorophyll-a responses (n=3 biological replicates), data points indicate responses for individual bottles, vertical lines indicate the range. C=Control, no added nutrients; N=1 μM NO_3 + 1 μM NH_4 addition; Fe=2 nM Fe addition; NFe=combined N and Fe addition.

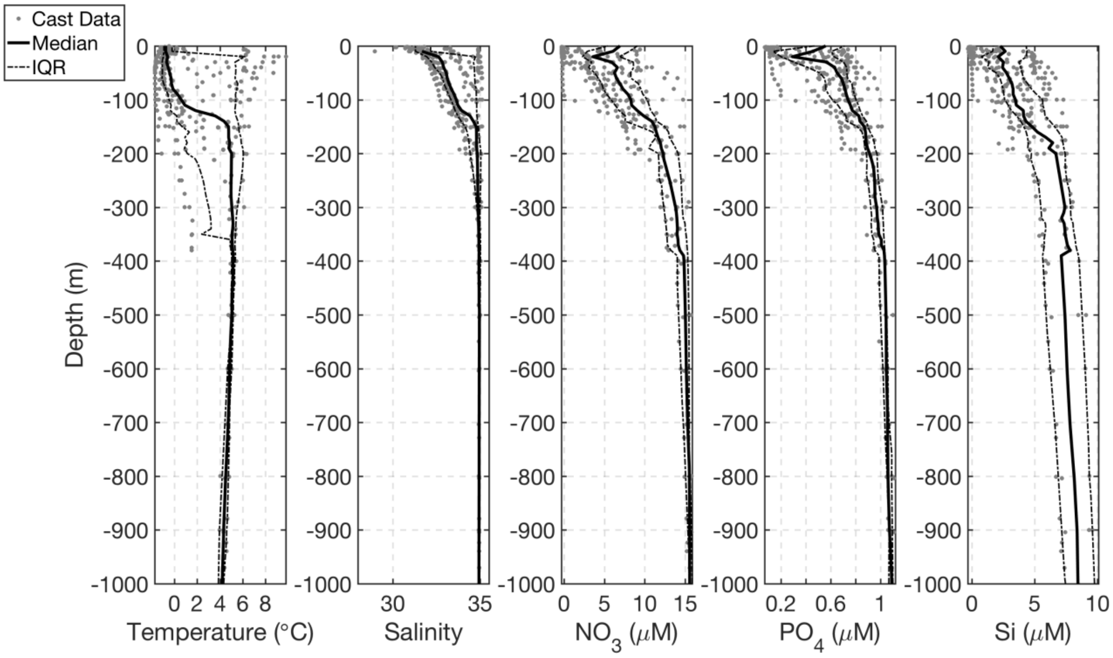

Supplementary Figure 2 Summer (June-July-August) macronutrient profiles used as input for the subglacial discharge plume model. Nutrient concentrations (open circles, as per Table S2) with median (thick black lines) and lower/upper quartiles (black dashed lines) shown.

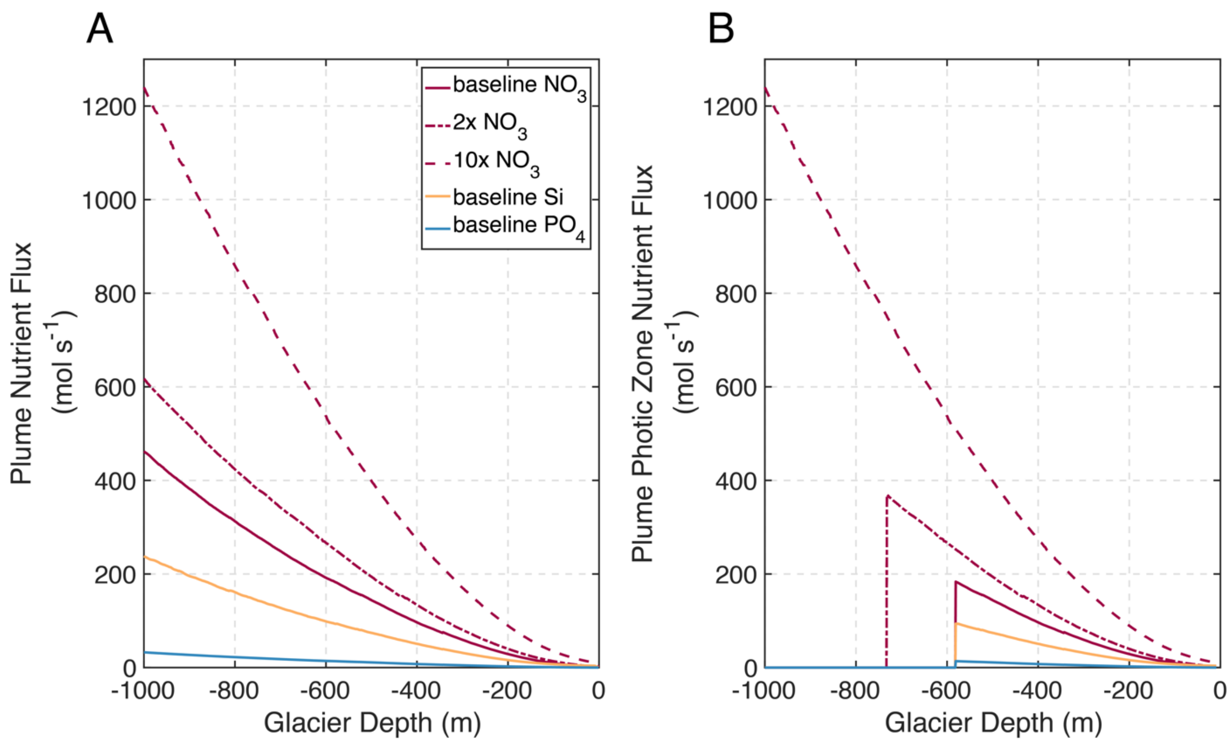

Supplementary Figure 3 Subglacial discharge plume model output. A 500 $m^3 s^{-1}$ discharge scenario (continuous lines for NO_3, PO_4 and Si) is plotted showing the plume macronutrient (NO_3, Si, PO_4) fluxes at the terminal depth (**A**) and into the photic zone (0-50 m) (**B**). Additional scenarios, with 2-fold (1000 $m^3 s^{-1}$) and 10-fold (5000 $m^3 s^{-1}$) higher subglacial discharge scenarios are also shown for NO_3 only (dashed lines). Note that for the 2× and 10× subglacial discharge scenarios, the resulting plume equilibrates within the photic zone at deeper glacier grounding line depths.

Cruise/ship	Year/Season	Nutrient data	Stations	Region	Project funding
RRS James Clark Ross JR302	June-July 2014	Unpublished, provided by Sinhue Torres-Valdes	47-50, 52, 53, 63, 64, 77-82, 108, 109	South Greenland	NERC RAGNARoCC (NE/K00249X/1)
RRS Discovery D354	July-August 2010	Unpublished, provided by Eric Achterberg	10-14	Southeast Greenland	NERC IBIS (NE/E006833/1)
FS Polarstern PS100, GN05	July-September 2016	Unpublished, provided by Kai-Uwe Ludwichowski and Martin Graeve	18-20	Northeast Greenland	DFG, GRIFF

Supplementary Table 1 Data source of shelf macronutrient profiles taken around Greenland. Station names are as per the respective cruise reports.

NO_3 / µM	DFe / nM	Fe* / nM	Latitude (°N)	Longitude (°E)
0.87	1.60	1.54	79.569	-19.518
0.87	1.36	1.30	79.569	-19.518
0.06	1.38	1.38	79.550	-18.688
0.06	4.12	4.11	79.550	-18.688
0.06	1.07	1.06	79.550	-18.688
0.06	2.13	2.13	80.149	-17.381
0.06	2.33	2.32	80.149	-17.381
1.27	1.15	1.06	79.196	-17.105
0.30	0.95	0.93	79.638	-16.656
0.00	1.00	1.00	77.867	-14.801
0.00	1.02	1.02	77.867	-14.801
0.00	0.84	0.84	77.867	-14.801
0.00	0.80	0.80	76.802	-8.615
0.00	0.70	0.70	76.802	-8.615
0.00	1.31	1.31	80.219	-8.144
0.27	1.67	1.65	78.678	-7.948
0.27	1.69	1.67	78.678	-7.948
0.41	1.77	1.74	79.589	-4.772
0.57	2.37	2.33	80.326	-4.009
0.37	0.48	0.45	78.804	-3.510
0.03	0.28	0.28	78.854	-2.567
0.07	0.39	0.39	78.822	-2.042

Supplementary Table 2 Surface NO_3 and dissolved Fe (DFe) concentrations determined on GEOTRACES section GN05 (used to calculate Fe*, Fig 1).

Glacier	Grounding line depth / m	Entrainment factor
Upernavik	100	4.1
Umiamako	230	19
Kangiata Nunata Sermia	250	12
Kangerdlugssup Sermerssua	250	16
Heilprin	350	34
Store	500	26
Tracy	610	81
Helheim	650	34
Kangerdlugssuaq	650	29
Jakobshavn Isbrae	800	29
Alison	850	57
Rink Isbrae	850	72

Supplementary Table3 Entrainment factors calculated at the plume terminal level for different glacier systems using data presented in Carroll et al.,[3]. Entrainment factors were weighted over the year by multiplying daily entrainment factors by the fraction of annual discharge occurring on each day.

Supplementary References

1. Browning, T. J., Bouman, H. A. & Moore, C. M. Satellite-detected fluorescence: Decoupling nonphotochemical quenching from iron stress signals in the South Atlantic and Southern Ocean. *Global Biogeochem. Cycles* **28,** 510–524 (2014).
2. Ryan-Keogh, T. J. *et al.* Spatial and temporal development of phytoplankton iron stress in relation to bloom dynamics in the high-latitude North Atlantic Ocean. *Limnol. Oceanogr.* **58,** 533–545 (2013).
3. Carroll, D. *et al.* The impact of glacier geometry on meltwater plume structure and submarine melt in Greenland fjords. *Geophys. Res. Lett.* **43,** 9739–9748 (2016).
